# Research highlights, *The Plant Genome*, Volume 13, Issue 3

**DOI:** 10.1002/tpg2.20068

**Published:** 2020-11-20

## 1 ALLELE SHIFTS IN IMPROVED WHEAT

Selection is the deliberate act of increasing the frequency of favorable alleles in a genome thereby affecting the phenotypic performance of the organism. Breeding for high yielding and disease resistant wheat cultivars has a long history in the United States. Ayalew et al. (https://doi.org/10.1002/tpg2.20032) identified significant selection signatures caused by several decades of wheat breeding in the Great Plains. The major selection signatures identified were connected to a translocation segment inherited from goatgrass (*Aegilops ventricosa*), a wild relative of wheat. Introducing genetic variants from distant relatives provides lasting solutions to some biotic and abiotic stresses.

## 2 ON HADAMARD AND KRONECKER PRODUCTS IN COVARIANCE STRUCTURES FOR GENOTYPE × ENVIRONMENT INTERACTION

Genomic prediction models including genotype × environment (G×E) interactions play an important role in plant breeding. In particular for multi‐environment trials, predictions can be used to reduce the testing effort by not planting each line at each location, but by predicting unobserved combinations (“sparse testing”). Two different mathematical matrix operations ‐ the Hadamard and the Kronecker product ‐ have been used in literature to define the covariance structure of interaction effects. Martini et al. (https://doi.org/10.1002/tpg2.20033) show that when appropriately used, both approaches lead to exactly the same covariance model, but the Kronecker formulation may have some computational advantages when inverting the matrix. Moreover, the results illustrate that a simple estimate of the environmental covariance can be obtained by the phenotypic covariance of the training set across environments. To achieve a high increase in predictive ability, each line should be at least observed in one environment and an estimate on the environmental covariance should be given.

## 3 GENOME PREDICTION OF WHEAT QUALITY TRAITS

Wheat quality improvement is important in all wheat‐breeding programs but due to the cost, time, and quantity of seed required, wheat quality is in the last stages of the breeding cycle with limited sample size. Genomic prediction could help to select wheat quality lines more efficiently (reducing the cost and time). In this research, Ibba et al. (https://doi.org/10.1002/tpg2.20034) evaluated the prediction performances of 13 wheat quality traits under two statistical models and where lines in the second year were predicted using the quality information in the first year. For most quality traits moderate to high prediction accuracies were found, suggesting that the use of genomic‐enabled prediction is feasible.

## 4 COMBINED STRESS TOLERANCE IS POSSIBLE

Das et al. (https://doi.org/10.1002/tpg2.20035) report that maize cultivars tolerant to individual stresses have been successfully developed, however, the major challenge lies in putting together tolerance to multiple abiotic stresses and develop cultivars with combined stress tolerance. Multi‐parent population involving trait donors for targeted stresses along with elite lines improved using rapid cycle genomic selection resulted in gains across stresses without yield penalty under optimal conditions. The approach also helped to shorten the breeding cycle and reduce the costs of phenotyping, and thereby increased genetic gains in terms of both cost and time.

## 5 LOCI REGULATE JUJUBE QUALITY TRAITS

Fruit size, stone size, and fruit cracking can affect commercial value of jujube fruit. Hou et al. (https://doi.org/10.1002/tpg2.20036) report that 45 significant markers associated with nine fruit quality traits. A total of 4651 high‐quality single nucleotide polymorphisms were identified in 180 jujube accessions using genotyping‐by‐sequencing. Three distinct groups, and rapid linkage disequilibrium decay was observed in this jujube population. Twenty‐one candidate genes involved in plant growth and development were identified from the genome sequences of jujube. These marker loci and their linked genes would provide a basis for marker‐assisted breeding and genomic selection to improve jujube cultivars.

## 6 RESISTANT SOYBEANS UNMASK INVISIBLE INVADER

Brown stem rot (BSR), caused by the *Phialophora gregata*, reduces soybean yield by up to 38%. Initially, the fungus spreads throughout the plant causing no visible symptoms but after several weeks it initiates a killing spree in susceptible lines. It is unclear when or how resistance occurs. McCabe and Graham  (https://doi.org/10.1002/tpg2.20037) used whole‐genome expression analyses to demonstrate BSR‐resistant lines initiate resistant responses hours after infection. Exploiting the genes and networks described will enable the development of novel diagnostic tools to facilitate breeding and screening for BSR resistance.

## 7 VITAMIN E‐RICH BARLEY SEEDS

Seed viability is a key component of the barley malting process that impacts beer production. Mahalingam et al. (https://doi.org/10.1002/tpg2.20039) report that there is extensive natural variation in the seed Vitamin E content of barley, a key factor in seed longevity and viability. Vitamin E, also called tocochromanol, comes in eight different variations and only barley seeds have all eight isoforms. The concentration of these isoforms was quantified in seeds from wild and cultivated barley lines via mass spectrometry. The genotypic variation among these lines was determined via array and sequencing technologies. The correlation between the genotypic variation and the phenotypic variation in the tocochromanol isoforms were examined with a statistical tool called genome‐wide association analysis. Genetic markers significantly associated with tocochromanol isoforms were identified and, interestingly, these markers were near every gene involved the Vitamin E biosynthesis pathway. This information will be useful for breeding programs for improving seed longevity, germination, and quality.

## 8 CORN SILK EMERGENCE TRIGGERS REPROGRAMMING

Corn silks are botanically extraordinary for their fast rate of growth, long length, and ability to remain viable for pollen reception while exposed to harsh conditions. Despite their unique biology and importance in crop production, silks have been the subject of few genomic studies. McNinch et al. (https://doi.org/10.1002/tpg2.20040) report that while transcriptomic variation among adjacent silk sections within the husk leaf encasement is modest, comparison across the emergence boundary reveals that exposure to the external environment leads to strong reprogramming of gene expression, particularly through activation of abiotic and biotic stress responses. Major expression trends were consistent between the two corn lines studied, although genotypic expression variability was documented for thousands of genes. This paradox reflects how the essence of silk function is overlaid with variation captured by breeding.

## 9 REFERENCE GENOME SEQUENCE OF *SALVIA MILTIORRHIZA*

*Salvia miltiorrhiza* Bunge, also known as red sage or Danshen, is an important traditional Chinese medicine used for thousands of years to treat cardiovascular and other diseases. Song et al. (https://doi.org/10.1002/tpg2.20041) reported a high‐quality reference genome of *S. miltiorrhiza* was generated by combining PacBio long read sequencing and chromatin interaction mapping (Hi‐C) technologies, resulting in chromosome‐scale assembly of a 594.75‐Mb genome sequence with a contig N50 of 2.70 Mb. The *S. miltiorrhiza* genome contained 32,483 protein‐coding genes, with a repetitive DNA content of approximately 64.84%. The high percentage of young LTRs suggests that multiple TE transposition bursts occurred recently in *S. miltiorrhiza*. Genes unique to secondary metabolism pathways were expanded in the *S. miltiorrhiza* genome. A new CYP450 gene cluster was identified in the phloem of red roots where active components were synthesized. This reference genome sequence will facilitate future studies on elucidation of secondary metabolism synthesis pathway and genetic improvement of *S. miltiorrhiza*.

## 10 ARTIFICIAL INTELLIGENCE UNRAVELING THE ENZYME'S FUNCTION

Enzyme reactions do not strictly represent metabolic pathways. Many bioinformatics tools use sequence similarity to assign enzyme reactions. Thus, defining the enzyme's functions is not a trivial task. Almeida and Valente (https://doi.org/10.1002/tpg2.20043) developed the mApLe program, a tool that uses artificial intelligence to predict the metabolic pathways of plants' enzymes without the use of sequence similarity or annotation transfer. mApLe can then accurately classify a massive diversity of enzymes, even those unknown or without any homolog. The user‐friendly interface can be installed in conventional computers.

## 11 GENETIC FACTORS INFLUENCE FLOWERING TIME

Early maturity of alfalfa can produce high quality hay with high feeding values. Flowering time is a key factor for measuring plant maturity and is genetically controlled. Zhang et al. (https://doi.org/10.1002/tpg2.20045) report that 85% of the phenotypic variance of flowering time is controlled by genetic factor. A total of 28 quantitative trait loci (QTL) were associated with flowering time. Eleven QTL have larger effects than others and they were located on two linkage groups. Thus, flowering time may be controlled by a few key genes with major effects and several minor genes. Further investigation of the key genes may help in regulating the flowering time of alfalfa plants.

## 12 PAN‐GENOME IN NONCODING RNA ANALYSES

Noncoding RNAs evolved from transposable elements and perform vital roles in the structural organization and functions of plant genomes. Transposable elements create genomic variation within a species that cannot be captured by a single reference genome, which in turn affects noncoding RNA analyses. Tahir ul Qamar et al. (https://doi.org/10.1002/tpg2.20046) explain that pan‐genomes can capture the entire genomic content and variation of a species and help to explore functions of noncoding RNAs for accelerated crop improvement. They summarize the recent progress in plant pan‐genomics and present a perspective about the origin of noncoding RNAs from transposable elements and how pan‐genomes could be helpful to explore and exploit noncoding RNAs.

## 13 GENOMIC MODEL PREDICTED APPLE COLORATION

Apple skin coloration is controlled by one or a few major genes/QTLs and affected by a set of minor‐effect QTLs. MdMYB1, located on chromosome 09, has repeatedly been reported as a major gene controlling apple fruit cover color. Zheng et al. (https://doi.org/10.1002/tpg2.20047) report identification nine minor‐effect QTLs on chromosomes 05, 08, 10, 11, and 15 for the degree of apple fruit cover color beyond MdMYB1. From these QTLs, ten candidate genes were predicted, including MdMYB1, and KASP markers were designed. The genotype effect and allele substitution effect of the markers were estimated. Finally, a non‐additive QTL‐based genomics assisted prediction model was established for the degree of apple fruit cover color. The Pearson's correlation coefficient between the genomic predicted value and the observed phenotype value was 0.5690. These results can be beneficial for apple genomics‐assisted breeding and may provide insights for understanding the mechanism of fruit coloration.

## 14 INTEGRATING GREENHOUSE PHENOTYPING AND GENOMIC SELECTION

The integration of genomic selection and early phenotyping can accelerate forest tree breeding. Alves et al. (https://doi.org/10.1002/tpg2.20048) show that the combination of rapid greenhouse phenotyping and genomic selection methodology can significantly increase the selection accuracy and genetic gains in forest tree breeding. Their results show that combining early phenotyping and genomic selection can increase response to selection by as much as 33% relative to conventional mass phenotypic selection.

## 15 EPIGENETIC LANDSCAPE OF SPRING BARLEY

DNA methylation is an epigenetic mark that regulates multiple processes, such as gene expression and genome stability, and its patterns vary widely among plant species. Malinowska et al. (https://doi.org/10.1002/tpg2.20049) developed a pipeline where regions with different methylation statuses were shared within the analyzed set of barley cultivars. A significant correlation between genetic and epigenetic distances suggests that a considerable portion of methylated regions is under strict genetic control in barley.

## 16 EMERGING STEM RUST RESISTANCE MAPPED

Stem rust is a devastating disease of wheat that can cause complete yield loss. Genetic resistance has been used to control this disease, but new emerging races of the wheat stem rust pathogen keep defeating resistance genes deployed in wheat cultivars. Edae and Rouse (https://doi.org/10.1002/tpg2.20050) identified and mapped resistance in elite North American wheat germplasm effective to the latest and most virulent wheat stem rust pathogen races. The association mapping techniques utilized allowed for the identification of resistance specific to groups of races, specific to particular temperatures, and specific to individual isolates of the same race. The resistance identified was largely race‐specific highlighting the need for testing of the latest races.

## 17 ENHANCING GENETIC VARIATION IN WHEAT

The USDA‐ARS wheat core collection is a rich source of useful genetic variation, but is underutilized because of the time and effort needed to introgress many genetic variants into elite backgrounds. To address this need, Sallam et al. (https://doi.org/10.1002/tpg2.20051) developed the Spring Wheat Multiparent Introgression Population (SWMIP), consisting of 2,038 recombinant inbred lines derived by crossing 25 diverse accessions of the core collection to the widely adapted Minnesota spring wheat cultivar RB07. The SWMIP and its parents were genotyped using genotyping‐by‐sequencing and phenotyped for heading date, height, test weight, and grain protein content. Genome‐wide association mapping was performed on the population and identified 16, 15, 12, and 13 marker‐trait associations (MTAs) for heading date, height, test weight, and grain protein content, respectively. Some of these MTAs were coincident with major genes known to control the traits, but others were novel and contributed by the genetically diverse core parents. The SWMIP will be a valuable source of genetic variation for spring wheat breeding.

## 18 PISTIL ABORTION IN JAPANESE APRICOT

Japanese apricot is an important fruit and ornamental plant with great economic value. In Japanese apricot, pistil abortion commonly occurs and causes severe losses to the production industry. Iqbal et al. (https://doi.org/10.1002/tpg2.20052) report that a transcription factor TCP4 is strongly involved in pistil abortion and in their study used ChIP‐Sequencing method to identify its downstream genes and peak related to this issue. They found some peak‐associated genes actively involved in this process and put forward the regulatory mechanism of PmTC4 during the pistil abortion process.

## 19 EVOLUTION OF SVP GENES IN ROSACEAE

MADS‐box genes that are homologous to Arabidopsis SHORT VEGETATIVE PHASE (SVP) have been shown to play key roles in the regulation of bud dormancy in perennial species, particularly in the deciduous fruit trees of Rosaceae. In this study, Liu et al. (https://doi.org/10.1002/tpg2.20053) found SVP genes to be significantly expanded in Rosaceae in a lineage‐specific manner, and which were extensively contributed by lineage‐specific gene duplication (GD) events. Interestingly, tandem duplications, a well‐known evolutionary feature of SVP genes, were found to be mainly Amygdaloideae‐specific. The lineage‐specific evolutionary profiles might be related to large functional innovations and diversification of SVP genes in Rosaceae.

## 20 ACCELERATING THE DEVELOPMENT OF VERTICILLIUM WILT RESISTANT STRAWBERRY CULTIVARS

Verticillium wilt is a soil‐borne fungal disease affecting more than 300 species of plants including strawberry. This disease threatens strawberry production worldwide. Pincot et al. (https://doi.org/10.1002/tpg2.20054) estimated the long‐term genetic gain in breeding for resistance to Verticillium wilt in strawberry. They found that genetic gains were either flat or negative and that a high frequency of cultivars developed over the last 165 years are susceptible but discovered several exotic sources of resistance. The latter included wild ecotypes and heirloom cultivars that are predicted to carry favorable alleles not found in modern cultivars. They further showed that genomic selection was a sound and accurate approach for identifying resistant individuals and accelerating the development of resistant cultivars. The latter can be achieved by shortening selection cycles, increasing throughput without phenotyping, non‐destructive testing (genotyping non‐infected plants), and selection in early developmental stages.

## 21 ETHIOPIAN SORGHUM HARBORS UNIQUE GENETIC DIVERSITY

Understanding population genetic structure and/or diversity of a crop, and defining genomic regions associated with important traits is essential for efficiency and success of crop improvement endeavors. Girma et al. (https://doi.org/10.1002/tpg2.20055) reported considerable genetic and phenotypic diversity in an Ethiopian sorghum landrace collection. These genetic resources capture a large diversity of morphological traits, tolerance to abiotic stress, and rare natural variants. In spite of these resources, and the enormous potential for introgression of unique variation, narrow genetic diversity was detected among improved sorghum varieties in Ethiopia, highlighting the need to broaden the germplasm foundation used as parental lines in breeding pipelines. A subset of the landrace collection was selected to capture the maximum diversity in the broader Ethiopian sorghum germplasm. Girma et al. further discovered genomic regions and candidate genes underlying sorghum adaptation to abiotic stresses.

## 22 PRACTICAL GENOMIC SELECTION APPROACH IN POTATO

Using genomic information to predict phenotypes can improve the accuracy of estimated breeding values and can potentially increase genetic gain over conventional breeding. Sood et al. (https://doi.org/10.1002/tpg2.20056) report an approach to economize genomic selection application in potato by combining the information of genotypes and non‐genotyped individuals to estimate breeding values using genomic best linear unbiased prediction. The prediction accuracies of the non‐genotyped progenies varied from 0.17‐0.70. The approach is especially attractive for a breeding program to get the most from its non‐genotyped historical germplasm, while only a small number of samples are genotyped.

## 23 POLYPLOID GENE EXPRESSION RESPONDS TO STRESSES

Many crop plants are polyploids and understanding how duplicated genes in polyploids respond to abiotic stresses is an important topic in crop science. Lee and Adams (https://doi.org/10.1002/tpg2.20057) report that duplicated genes in polyploid *Brassica napus* showed considerable changes in expression and alternative splicing in response to abiotic stresses, with cold stress showing the greatest responses. Expression and alternative splicing were negatively correlated in responding to the stresses. The divergence of gene expression and alternative splicing patterns in duplicated genes may increase the flexibility of polyploids in responding to various environmental stresses.

## 24 LIGHT REGULATES CUTICULAR WAX DEPOSITION

Leaf cuticular waxes are sensitive to changing environments. Huang et al. (https://doi.org/10.1002/tpg2.20058) discovered that cuticular waxes of faba bean leaf were sensitive to light wavelengths. Yellow light significantly increased the total wax coverage and wax crystal density of faba bean leaf. Light wavelengths changed the relative abundance of dominant primary alcohol from C_24_ under white, yellow, and red lights to C_26_ under blue and purple lights. The candidate waxy genes selected by RNA‐seq and transcriptome processing were also differentially regulated by different light wavelengths. Such changes of the physical and chemical properties of leaf cuticular wax induced by light wavelengths also altered its eco‐physiological functions, such as cuticle transpiration. Light wavelengths could also be used as a method for screening wax biosynthesis candidate genes.

## 25 ANALYSIS OF SUCROSE CONCENTRATION

The sucrose concentration in soybean seed significantly affects the flavor of soybean‐derived products. Sui et al. (https://doi.org/10.1002/tpg2.20059) conducted a genome‐wide association analysis based on 178 elite accessions and 33,149 single‐nucleotide polymorphisms. A total of five QTNs that related sucrose concentration were detected. Haplotypes of candidate genes significantly associated with sucrose concentration were identified. Ten candidate genes associated with sucrose concentration were predicted in soybean seeds.

## 26 MAPPING LEAF RUST RESISTANCE IN WHEAT

Constant evolution of new and virulent *Puccinia triticina* races worldwide demands the continuous search for novel sources of leaf rust resistance. In this study, Sapkota et al. (https://doi.org/10.1002/tpg2.20061) developed a recombinant inbred line (RIL) population consisting of 225 lines, genotyped the RIL population with the Illumina iSelect 90K SNP marker array, and constructed a high‐density linkage map. Furthermore, they mapped and characterized a novel and important adult plant leaf rust resistance gene *Lr2K38* in soft red winter wheat cultivar AGS 2038. The marker linked to *Lr2K38* was validated on a set of wheat germplasm and should be useful for selecting the leaf rust resistance in wheat breeding programs.

## 27 SEQUENCE ANALYSIS OF WHEAT SUBTELOMERES

Sequence analysis of wheat subtelomeres reveals a high polymorphism among homoeologous chromosomes. Aguilar and Prieto (https://doi.org/10.1002/tpg2.20065) report a detailed molecular analysis of wheat subtelomeres and showed a high polymorphism in the subtelomeric region among homoeologues (equivalent chromosomes from related genomes) for all the features analyzed, including genes, transposable elements, repeats, GC content, predicted CpG islands, recombination hotspots, and targeted sequence motifs for relevant DNA‐binding proteins. These polymorphisms might be the molecular basis for the specificity of homologous recognition and pairing in initial chromosome interactions at the beginning of meiosis in wheat.

